# The Emerging Roles of GlycoRNAs in the Pathogenesis of Sepsis

**DOI:** 10.3390/cells15030275

**Published:** 2026-02-01

**Authors:** Xiang Li, Saichaitanya Nallajennugari, Joshua Fu, Anfal Faisal, Mingui Fu

**Affiliations:** Department of Biomedical Science, Shock/Trauma Research Center, School of Medicine, University of Missouri Kansas City, Kansas City, MO 64108, USA; xlkgk@umkc.edu (X.L.); srnnkz@umkc.edu (S.N.); joshua.fu@ku.edu (J.F.); anfalfaisal613@gmail.com (A.F.)

**Keywords:** glycoRNAs, sepsis, inflammation, pathogenesis, immune cells

## Abstract

Sepsis is a life-threatening condition caused by a dysregulated host immune response to infection, leading to systemic inflammation, organ dysfunction, and potentially death. Despite significant advances in understanding the pathophysiology of sepsis, effective therapeutic options remain limited, and mortality rates remain unacceptably high. Therefore, a deeper understanding of sepsis pathogenesis and the identification of novel therapeutic targets are urgently needed to improve patient outcomes. Recent studies have revealed that RNAs can undergo glycosylation, generating a previously unrecognized class of molecules known as glycosylated RNAs (glycoRNAs), which are localized on the outer surface of cells. GlycoRNAs are highly expressed in immune cells, and accumulating evidence indicates that they play important roles in regulating immune responses, including immune cell adhesion and infiltration, immune cell activation, and immune evasion. In addition, glycoRNAs are abundantly expressed on the epithelial cell surfaces of the respiratory, digestive, urinary, and reproductive systems, suggesting that glycoRNAs may function as a component of epithelial barriers that protect against pathogenic invasion. Collectively, these findings suggest that glycoRNAs may play a critical role in the pathogenesis of sepsis. This review summarizes the expression and functions of glycoRNAs in immune and barrier systems and highlights their potential roles during distinct immunological phases of sepsis.

## 1. Introduction

Immunity serves as the primary defense against invading pathogens. Under physiological conditions, a balanced and tightly regulated immune response enables the host to efficiently eliminate pathogens and damaged cells without causing significant injury to its own tissues. In sepsis, however, the normal negative feedback mechanisms that terminate inflammation fail, resulting in uncontrolled hyperinflammation and subsequent organ damage [1,2,3]. Compensatory responses aimed at restoring immune homeostasis are often excessive, leading to immunosuppression or immune paralysis [4,5]. These two dysregulated immune phases frequently overlap and coexist, which likely explains why many immunomodulatory therapies for sepsis have failed in clinical trials [6,7,8]. Restoring immune balance therefore represents a central challenge in the treatment of sepsis. A deeper understanding of the regulatory mechanisms governing immune responses during sepsis is essential for the development of personalized and targeted therapeutic strategies.

Traditionally, RNAs have been viewed as molecules that function exclusively within the nucleus and cytoplasm of mammalian cells. Recent advances, however, have uncovered a surprising dimension of RNA biology: the presence of specific RNA species on the outer surface of mammalian cells [9,10]. One of the most intriguing discoveries is the identification of glycosylated RNAs (glycoRNAs), which are small noncoding RNAs covalently modified with complex N-glycans. Although glycoRNAs are synthesized intracellularly, they are transported to and displayed on the cell surface [10]. While their precise biological functions remain incompletely understood, emerging evidence suggests that glycoRNAs play important roles in cell–cell communication, pathogen-host interaction and immune regulation [11,12,13,14,15,16,17]. This is supported by several observations: (1) glycoRNAs are highly expressed in immune cells and immune organs [11,12]; (2) cell surface glycoRNAs mediate neutrophil and monocyte adhesion and infiltration through selective interactions with P-selectin and Siglec-5 on endothelial cells [13,14,15]; and (3) modified small nuclear RNAs (snRNAs) and their associated proteins, such as histones, present on the cell surface can trigger inflammatory responses [16,17].

Given that dysregulated host immunity is the core pathological feature of sepsis [7], glycoRNAs—novel immunomodulatory molecules displayed on the cell surface—are highly likely to participate in sepsis pathogenesis. In this review, we summarize recent advances in the study of glycoRNA expression and function and highlight their potential roles in the development and progression of sepsis.

## 2. The Effective Treatments for Sepsis Remain Limited

Sepsis is a life-threatening organ dysfunction caused by a dysregulated host response to infection and represents one of the leading causes of mortality in intensive care units worldwide [7]. Despite considerable advances in the understanding of its pathophysiological mechanisms over recent decades, clinical mortality rates associated with sepsis remain persistently high. This underscores the substantial limitations of existing effective treatments and highlights a significant unmet medical need [8]. The current standard clinical management of sepsis primarily relies on early infection control—including prompt administration of broad-spectrum antibiotics and source control—coupled with organ support strategies such as fluid resuscitation, vasoactive drug administration, mechanical ventilation, and symptomatic management [6]. Although these supportive measures can temporarily stabilize a patient’s vital signs, they often fail to reverse the core pathological processes driving sepsis. The repeated failure of targeted therapies directed at single inflammatory mediators—such as anti-endotoxin antibodies and anti-TNF-α antibodies—in clinical trials further confirms the complexity of sepsis pathogenesis. Rather than being solely driven by an exaggerated inflammatory response, sepsis involves a dynamic interplay of immune dysregulation, endothelial barrier injury, coagulopathy, and metabolic and cellular dormancy [18]. A deeper understanding of this intricate pathophysiological network has redirected therapeutic development toward more integrated strategies. Consequently, the current research focus has shifted from merely suppressing inflammation to multi-target modulation, with immunomodulation, pathogen clearance, endothelial stabilization, and mitochondrial protection emerging among the most promising therapeutic avenues [19,20]. Further investigation into the understanding of sepsis pathogenesis and the identification of novel therapeutic targets hold significant potential for improving clinical outcomes in sepsis.

## 3. GlycoRNAs Is a Group of Newly Discovered Biomolecules

The conventional view holds that the cell surface is primarily composed of biomolecules such as glycoproteins, glycolipids, and polysaccharides, which play central roles in intercellular recognition, signal transduction, and inflammatory responses. In contrast, RNA has traditionally been considered to reside exclusively within the nucleus and cytoplasm. This long-standing paradigm was overturned in 2021 by Flynn et al., who demonstrated that RNA could undergo glycosylation, giving rise to a previously unrecognized class of biomolecules termed glycosylated RNAs (glycoRNAs). Their study showed that glycoRNAs are synthesized intracellularly and subsequently displayed on the outer surface of the plasma membrane in a wide range of cell types [10]. These glycoRNAs consist predominantly of small noncoding RNAs—including small nuclear RNAs (snRNAs), small nucleolar RNAs (snoRNAs), transport RNAs (tRNAs), microRNAs (miRNAs), ribosome RNAs (rRNAs) and Y RNAs—that are modified by complex N-glycans. Notably, the glycan structures are highly sialylated and fucosylated, closely resembling those found on glycoproteins [10]. GlycoRNA biosynthesis depends on the canonical intracellular N-glycosylation pathway, followed by trafficking to the cell surface by an unknown mechanism.

The molecular characteristics of glycoRNAs remain incompletely defined. A recent study profiled glycoRNA species across 13 human cell lines using rPAL-seq [21]. Surprisingly, unsupervised clustering based on global glycosylation patterns segregated these cell lines into two major groups that broadly corresponded to hematopoietic and non-hematopoietic lineages. Most glycoRNAs were found to be cell line-specific; however, a subset of “core” glycoRNAs was recurrently detected in at least 75% of the cell lines examined, along with a smaller group of universally present glycoRNAs. When glycoRNAs were ranked by both recurrence and glycosylation abundance, the top 20 species consisted almost exclusively of snRNAs and snoRNAs, whereas tRNAs and rRNAs contributed predominantly to hematopoietic cell lines. Universally detected glycoRNAs were dominated by U-rich RNAs, particularly U snRNAs, with U1, U2, U4, and U3 among the most prominent species [21]. A separate study using distinct methodologies reported similar cell-surface RNA profiles, likewise, enriched for U-rich RNAs [22]. Despite these advances, it remains unclear whether diversity in RNA backbones confers functional differences among glycoRNAs in mediating cell–cell interactions and signal transduction. Notably, the discovery that acp3U serves as an attachment site for glycan chains on RNA represents a breakthrough [11]. Consistent with this finding, rPAL-seq data further suggests that modified uridines may act as preferential sites for glycosylation in glycoRNAs [21].

The compositions and structures of glycoRNA-associated glycans are cell type-specific and heterogeneous. Using Ac_4_ManNAz labeling, we identified two distinct forms of glycoRNAs—glycoRNA-L and glycoRNA-S—in THP1 cells [15]. Our results suggest that glycoRNA-L carries complex N-glycan structures containing sialic acids, N-acetylglucosamine, N-acetylgalactosamine, galactose, mannose, and fucose. In contrast, glycoRNA-S appears to be modified predominantly with N-acetylgalactosamine [15]. These findings indicate that most mammalian cells may harbor at least two populations of glycoRNAs defined by distinct glycoforms. Among human cell types, however, more than two glycoRNA populations are likely to present, as evidenced by the multiple bands observed in different tissues via glycoRNA Northern blot analyses [12]. Decoding the glycan structures of glycoRNAs in specific functional cell types will be essential for understanding their functional diversity and may facilitate the identification of novel clinical diagnostic biomarkers and therapeutic targets.

Importantly, emerging evidence indicates that glycoRNAs can selectively bind members of the sialic acid-binding immunoglobulin-like lectin (Siglec) family of immune receptors. Siglecs are critical regulators of immune cell activation and tolerance, and dysregulation of Siglec signaling has been implicated in autoimmune diseases and inflammatory disorders [23,24,25]. As a newly identified class of endogenous Siglec ligands, glycoRNAs are therefore likely to play previously unappreciated roles in initiating and modulating intercellular communication and immune responses [26]. This discovery establishes a conceptual bridge between the historically distinct fields of RNA biology and glycobiology, opening a new frontier in immunological research.

In the context of sepsis—a disease characterized by systemic and dysregulated inflammatory responses—glycoRNAs may offer a novel framework for understanding the molecular mechanisms underlying immune dysfunction. Elucidating how glycoRNAs contribute to immune cell activation, regulation, and tolerance holds considerable promise for the development of innovative diagnostic biomarkers and immunomodulatory therapeutic strategies for sepsis.

## 4. Robust Expression of GlycoRNAs in Immune and Barrier Systems

Using three complementary methodological approaches—metabolic labeling of living cells or animals, RNA–periodate oxidation and aldehyde ligation (rPAL), which enables direct detection of native glycoRNAs, and lectin-based detection (LBD)—we systematically characterized the expression profiles of glycosylated RNAs across 24 human and mouse tissues [12]. This comprehensive analysis revealed a striking tissue-specific distribution of glycoRNAs. Immune organs emerged as the sites with the highest glycoRNA abundance, with particularly strong expressions detected in bone marrow, thymus, spleen, lymph nodes, and peripheral blood leukocytes. This enrichment strongly suggests that glycoRNAs play important roles in immune cell development, activation, and inflammatory responses. Notably, glycoRNAs were also highly expressed in barrier tissues that directly interface with the external environment, including the respiratory, digestive, urinary, and reproductive tracts. This distribution implies potential functions for glycoRNAs in mucosal immunity and in maintaining epithelial barrier defenses against pathogen invasion. Moderate levels of glycoRNA expression were observed in tissues such as the brain, heart, and white adipose tissue, although the physiological roles of glycoRNAs in these organs remain largely unexplored. In contrast, glycoRNA expression was minimal or undetectable in skin, skeletal muscle, liver, kidney, and brown adipose tissue. These highly specific expression patterns indicate that glycoRNAs are not ubiquitous housekeeping molecules but rather are selectively associated with core physiological processes such as immune surveillance and barrier maintenance. A deeper understanding of the tissue-specific expression and regulation of glycoRNAs will be essential for elucidating their functions in both health and disease. Such insights may also facilitate the evaluation of glycoRNAs as novel diagnostic biomarkers or therapeutic targets for sepsis.

## 5. GlycoRNAs Are Critical Players in the Regulation of Immune Response

### 5.1. GlycoRNAs Mediate Inflammatory Cell Adhesion and Infiltration

The pathogenesis of sepsis remains incompletely understood; however, excessive infiltration of hyperactivated immune cells, particularly neutrophils and monocytes, into sites of inflammation is widely recognized as a central event that exacerbates tissue injury during sepsis. This process depends on the tightly regulated migration of immune cells from the circulation to inflamed tissues and is mediated by complex intercellular interactions between immune cells and the vascular endothelium. Recent studies have identified glycoRNAs as key regulators of immune cell adhesion and trafficking. Zhang et al. demonstrated that glycoRNAs displayed on the surface of neutrophils can specifically recognize P-selectin expressed on activated endothelial cells, thereby directly promoting neutrophil recruitment to inflammatory sites. Importantly, preclinical studies showed that disruption of the glycoRNA–P-selectin interaction markedly reduced neutrophil infiltration in an acute lung injury model, providing compelling evidence that glycoRNAs are indispensable regulators of neutrophil migratory responses during inflammation [13].

Subsequent studies have revealed similarly important roles for glycoRNAs in monocyte biology. Our group identified two distinct glycoRNA subtypes on the surface of human monocytes—glycoRNA-L (long-chain) and glycoRNA-S (short-chain)—both of which can directly bind sialic acid-binding immunoglobulin-like lectin-5 (Siglec-5) expressed on endothelial cells. This interaction promotes monocyte adhesion to activated endothelial cells and suggests that these glycoRNA subtypes play critical roles in modulating monocyte–endothelial interactions [15].

Our recent study demonstrated that glycoRNAs are not expressed in resting endothelial cells but are robustly induced during endothelial activation by a range of inflammatory stimuli, including lipopolysaccharide (LPS), TNF, IL-1, IL-6, and homocysteine. Endothelial glycoRNAs were found to directly bind Siglec-5 or Siglec-14 expressed on leukocytes. These findings suggest that glycoRNA–Siglec interactions may represent a common mechanism mediating leukocyte–endothelial cell interactions during inflammation. Similarly, glycoRNA–Siglec interactions may facilitate the adhesion of cancer cells to endothelial cells and thereby influence cancer metastasis. Collectively, these observations imply glycoRNAs as important contributors to immune cell and cancer cell trafficking and, consequently, to the pathogenesis of sepsis and cancer metastasis [14].

### 5.2. GlycoRNAs May Mediate Immune Cell Activation and Recognition

Beyond these findings, one of the most striking emerging functions of glycoRNAs is their role in mediating communication between cells and the extracellular environment. RNA-binding proteins (RBPs) localized on the surface of living cells can form functional complexes with glycoRNAs, termed glycoRNA–csRBP clusters [27]. These clusters create binding platforms for cell-penetrating peptides, thereby facilitating their cellular entry via endocytosis or related mechanisms and modulating cell–environment interactions [27]. Notably, glycoRNAs also form clusters with cell surface-localized RBPs and heparan sulfate. Heparan sulfate is associated with specific secondary structures of glycoRNAs, not only stabilizing their anchorage at the cell surface but also recruiting immune receptors to promote immune cell activation [28]. Consistent with these observations, another study reported that glycoRNA–heparan sulfate clusters facilitate the recruitment of growth factors and subsequent cell activation [29]. Whether such glycoRNA clusters with RBPs and heparan sulfate contributes to cytokine-induced immune cell activation remains an important question for future investigation. Interestingly, Jiang et al. demonstrated that Y RNAs present on the surface of human monocytes enrich extracellular histones, thereby regulating interleukin-6 (IL-6) gene expression and protein secretion upon histone stimulation through NF-κB and AP-1 signaling pathways. These findings reveal a previously unrecognized immune activation mechanism mediated by surface-associated Y RNAs on monocytes [17]. In addition, when glycoRNAs bind to Siglec, they may trigger activating or inhibitory signaling in targeting cells and regulate the activating status of targeting cells. Overall, there may be multiple functions of glycoRNAs in regulating immune cell activation and recognition, which need to be further determined by in vitro and vivo evidence.

### 5.3. GlycoRNAs May Represent a Novel Mechanism of Immune Evasion

Beyond their role in mediating cell adhesion and trafficking, recent studies have uncovered a novel function of glycoRNAs in immune evasion and immune tolerance. Specifically, evidence indicates that the N-glycan moiety of glycoRNAs can shield the acp3U modification (3-(3-amino-3-carboxypropyl)uridine) on the associated RNA molecule, thereby preventing its recognition by the innate immune system as “non-self” and avoiding inappropriate inflammatory activation [16]. When glycoRNAs are enzymatically deglycosylated using glycosidases such as PNGase F, the exposed acp3U-modified RNAs become potent ligands for endosomal Toll-like receptors TLR3 and TLR7, leading to robust activation of the type I interferon pathway and induction of strong inflammatory responses. This glycan-mediated shielding mechanism plays an essential role during efferocytosis, ensuring that macrophages do not mount inflammatory responses upon encountering apoptotic cell-derived glycoRNAs, thereby preserving tissue homeostasis. Consistent with this model, genetic deletion of DTW-domain containing protein 2 (DTWD2), a key enzyme required for acp3U biosynthesis, completely abolishes the immunostimulatory activity of deglycosylated RNAs, confirming that acp3U is the critical molecular determinant for innate immune activation [16].

### 5.4. GlycoRNAs May Be Used as a Tool to Treat Inflammatory Diseases

Building on the regulatory roles of glycoRNAs in inflammation, Zhang and colleagues developed a novel nanoparticle-based therapy, termed glycoRNA-NP-siMT1. These nanoparticles incorporate glycoRNAs derived from HL60 cell membranes as core components, thereby conferring neutrophil-mimicking properties that enable their targeted accumulation within the abdominal aortic aneurysm (AAA) lesion microenvironment. Treatment with glycoRNA-NP-siMT1 competitively inhibited neutrophil infiltration, significantly reduced neutrophil abundance in AAA lesions, and attenuated the formation of neutrophil extracellular traps (NETs) [30]. Importantly, we recently observed that glycoRNAs can be cleaved from the cell surface and released into circulation during sepsis. These circulating glycoRNAs may act as proinflammatory mediators, contributing to the cytokine storm characteristic of early-stage sepsis. Accordingly, therapeutic strategies aimed at removing or neutralizing circulating glycoRNAs may represent a novel approach to dampening excessive inflammation and improving outcomes in sepsis.

## 6. GlycoRNAs May Serve as a Component of Barrier to Prevent Pathogen Invasion

Physiological barrier systems constitute the body’s first line of defense against pathogenic invasion, and their integrity is essential for maintaining internal homeostasis and preventing infection. Our studies have demonstrated that glycoRNAs are not only enriched on the surfaces of immune cells but are also abundantly expressed on epithelial cells that form key physiological barriers, including those of the respiratory, digestive, urinary, and reproductive systems [12]. This highly selective distribution suggests that glycoRNAs may represent a previously unrecognized functional component of the innate immune barrier. The barrier functions of glycoRNAs are likely mediated through multiple, non-mutually exclusive mechanisms. First, acting as “molecular antennas” on the cell surface, glycoRNAs may exploit terminal sialic acid residues and other glycan structures to competitively engage host or pathogen-binding receptors, thereby directly interfering with pathogen adhesion and internalization. This mechanism may be particularly relevant for viruses such as adenovirus and influenza virus. Supporting this notion, in cultured human airway epithelial cell models, selective removal of cell surface glycoRNAs by RNase treatment markedly increased adenovirus infection efficiency [31], personal communication). Second, cell surface glycoRNAs may organize into specialized microdomains together with RNA-binding proteins, potentially forming receptor-like platforms that can interact with specific pathogens, including viruses and bacteria. Third, epithelial glycoRNAs may engage immune regulatory receptors, such as members of the sialic acid-binding immunoglobulin-like lectin (Siglec) family [23], thereby contributing to “self” recognition signals that help distinguish host tissues from invading pathogens. This interaction may prevent excessive immune activation against self while facilitating appropriate immune responses to microbial invasion. In the context of sepsis, disruption of physiological barriers, particularly intestinal barrier dysfunction leading to bacterial and endotoxin translocation—is a pivotal event that precipitates systemic hyperinflammation and multiple organ failure. Therefore, elucidating the mechanisms by which glycoRNAs contribute to the maintenance of epithelial barrier integrity, especially within the gut, is of critical importance for advancing our understanding of sepsis pathogenesis and for developing novel barrier-protective therapeutic strategies.

## 7. Discussion

**Figure 1:**
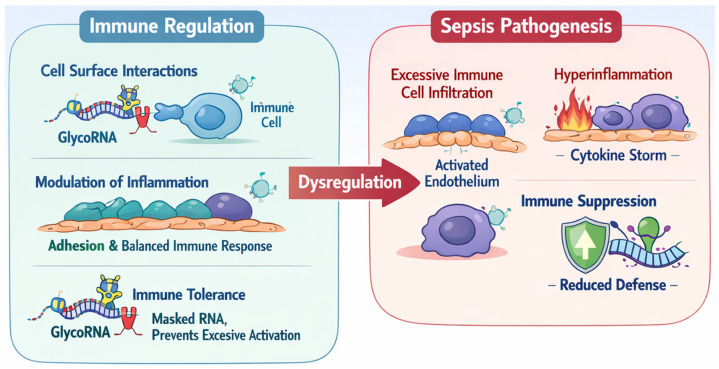
Schemic cartoon showing the roles of glycoRNAs in immune regulation and sepsis pathogenesis. Under physiological conditions, cell surface glycoRNAs mediate intercellular communication, facilitate immune cell adhesion and infiltration to combat infection, and mark endogenous RNAs to prevent inappropriate autoimmune responses. In the context of sepsis, dysregulated glycoRNA signaling may contribute to excessive hyperinflammatory responses during the early stages and to immune suppression during the later stages of the disease.

The discovery of glycosylated RNAs (glycoRNAs) has undeniably opened a new frontier in cell surface biology and immunology, representing a paradigm shift in our understanding of RNA function and glycan-mediated signaling. This review synthesizes emerging evidence positioning glycoRNAs as important regulators of immune homeostasis and barrier integrity, with significant implications for redefining the molecular pathogenesis of sepsis and for identifying novel therapeutic targets. Under physiological conditions, cell surface glycoRNAs mediate intercellular communication, facilitate immune cell adhesion and infiltration to combat infection, and mark endogenous RNAs to prevent inappropriate autoimmune responses. In the context of sepsis, dysregulated glycoRNA signaling may contribute to excessive hyperinflammatory responses during the early stages and to immune suppression during the later stages of the disease (Figure 1).

Despite these advances, most existing evidence is derived from in vitro studies, and the roles of glycoRNAs in sepsis remain largely inferred rather than directly demonstrated in vivo. There is therefore an urgent need to develop glycoRNA-deficient or glycoRNA-modulated animal models to rigorously evaluate the functions of glycoRNAs in immune regulation and to validate their contributions to sepsis pathogenesis. Such efforts will be essential for translating glycoRNA biology into clinically meaningful diagnostic and therapeutic strategies.

## 8. Research Gaps and Future Directions

**Figure 2:**
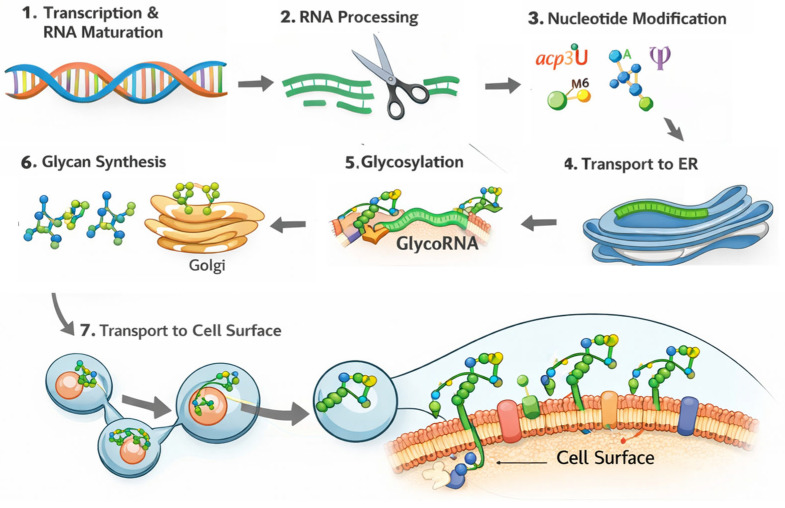
Schemic cartoon showing the potential glycoRNAs biosynthesis pathways. (1) Transcription and maturation of small non-coding RNA transcripts; (2) Processing of transcripts into short RNA fragments by unknown enzymes; (3) Nucleotide modifications within RNA fragments; (4) Transport of modified RNA fragments to the endoplasmic reticulum possible by Sidt1/Sidt2; (5) Enzymatic conjugation of glycans to RNA fragments in ER; (6) Glycan trimming in Golgi network and (7) Trafficking and display on the cell surface.

Despite its considerable potential, glycoRNA research remains in its infancy, and many fundamental scientific questions remain unresolved. One of the primary challenges lies in elucidating the biosynthetic and trafficking mechanisms of glycoRNAs. As hybrid molecules composed of RNA transcripts and glycan chains, glycoRNAs are generated through a multistep process that likely includes: (1) transcription and maturation of RNA transcripts; (2) processing of transcripts into short RNA fragments; (3) nucleotide modifications within RNA fragments; (4) transport of modified RNA fragments to the endoplasmic reticulum; (5) synthesis of glycan chains; (6) enzymatic conjugation of glycans to RNA fragments; and (7) trafficking and display on the cell surface (Figure 2). Current evidence suggests that glycoRNA biosynthesis depends on the convergence of classical N-glycan biosynthetic pathways and RNA metabolic processes [10]. However, the specific glycosyltransferase responsible for attaching the glycan moiety to the key acp3U nucleotide remains unidentified. Identifying and validating the enzyme catalyzing this critical step will be essential for developing precise tools to manipulate glycoRNA synthesis and function. In addition, how the RNA backbones of glycoRNAs are selected and processed is totally unknown. Uncovering these central mechanisms of glycoRNA biogenesis not only helps to generate specific glycoRNA-deficient animal models but also may provide targets for interfering with glycoRNA pathways. Another major unanswered question concerns the mechanism by which glycoRNAs are transported to the plasma membrane. Preliminary cellular studies suggest that Sidt1 and Sidt2—transmembrane double-stranded RNA (dsRNA) transporters—may play important roles in this process [32]. Future investigations using conditional gene knockout mouse models will be necessary to confirm the physiological relevance of these transporters in vivo.

Under the context of sepsis, all results currently obtained are from cell culture models. The evidence for the roles of glycoRNAs in the pathogenesis of sepsis is referring but not directly demonstrated. The lack of suitable animal models that faithfully recapitulate the complexity of human physiology represents a significant bottleneck in the field. Future efforts should focus on generating conditional knockout mouse models targeting key enzymes involved in glycoRNA biosynthesis (such as DTWD2) or transport machinery, as well as developing human disease-relevant systems, including organoid-based models and human immune cell transplantation approaches. These platforms will be critical for interrogating the roles of glycoRNAs in infection, inflammation, and sepsis.

Dynamic changes in leukocyte surface glycoRNA expression have been observed in both animal models and septic patients. In LPS- and cecal ligation and puncture (CLP)-induced sepsis, leukocytes exhibit decreased surface glycoRNA levels, accompanied by increased circulating glycoRNAs in the bloodstream. The role of circulating glycoRNAs in sepsis pathogenesis remains unclear, and the underlying mechanisms and functional significance warrant further investigation.

In parallel, continued innovation in glycoRNA detection technologies is urgently needed. During preparation of the manuscript, we are happy to see several methodologies with spatial resolution and multiplexing capacity to fully capture glycoRNA dynamics in complex tissues have been established [33,34,35,36,37]. The development of advanced imaging, single-cell, and high-throughput detection techniques will greatly enhance our ability to study glycoRNA localization, abundance, and functional interactions.

Beyond immune cell recruitment, future research should also explore the direct roles of glycoRNAs in epithelial innate immunity. For example, it remains determined whether glycoRNAs can directly interfere with pathogen adhesion through steric hindrance or receptor competition at barrier surfaces. Given their strategic localization on the cell surface, glycoRNAs represent highly attractive candidates as diagnostic biomarkers and therapeutic targets. Translational opportunities include the development of blood-based glycoRNA assays for early sepsis diagnosis and the exploration of diverse therapeutic strategies aimed at modulating glycoRNA signaling.

In summary, glycoRNA research stands at a critical transition point—from phenomenological description toward mechanistic dissection and functional application. Through interdisciplinary collaboration, systematic investigation of biosynthetic and transport mechanisms, and the development of innovative experimental models and technologies, the field is poised to significantly advance our understanding of this novel class of biomolecules. These efforts have the potential to yield transformative breakthroughs in the diagnosis and treatment of sepsis and other human diseases.

## Data Availability

No new data were created or analyzed in this study.

## Abbreviations

TLR, Toll-like receptor; CAR, coxsackie and adenovirus receptor; Siglec, sialic acid-binding immunoglobulin-like lectin; acp^3U^, 3-(3-amino-3-carboxypropyl)uridine; rPAL, RNA-optimized periodate oxidation and aldehyde ligation; LBD, lectin-based detection; DTWD2, DTW domain containing 2; GlycoRNA, glycosylated RNA.

## References

[1] Stearns-Kurosawa D.J., Osuchowski M.F., Valentine C., Kurosawa S., Remick D.G. (2011). The pathogenesis of sepsis. Annu. Rev. Pathol..

[2] Angus D.C., Linde-Zwirble W.T., Lidicker J., Clermont G., Carcillo J., Pinsky M.R. (2001). Epidemiology of severe sepsis in the United States: Analysis of incidence, outcome, and associated costs of care. Crit. Care Med..

[3] Bhan C., Dipankar P., Chakraborty P., Sarangi P.P. (2016). Role of cellular events in the pathophysiology of sepsis. Inflamm. Res..

[4] Chen Y., Guo D.Z., Zhu C.L., Ren S.C., Sun C.Y., Wang Y., Wang J.F. (2023). The implication of targeting PD-1:PD-L1 pathway in treating sepsis through immunostimulatory and anti-inflammatory pathways. Front. Immunol..

[5] Garduno A., Cusack R., Leone M., Einav S., Martin-Loeches I. (2023). Multi-omics endotypes in ICU sepsis-induced immunosuppression. Microorganisms.

[6] Wood K.A., Angus D.C. (2004). Pharmacoeconomic implications of new therapies in sepsis. Pharmacoeconomics.

[7] Singer M., Deutschman C.S., Seymour C.W., Shankar-Hari M., Annane D., Bauer M., Bellomo R., Bernard G.R., Chiche J.-D., Coopersmith C.M. (2016). The third international consensus definitions for sepsis and septic shock (Sepsis-3). JAMA.

[8] Nakamori Y., Park E.J., Shimaoka M. (2021). Immune deregulation in sepsis and septic shock: Reversing immune paralysis by targeting PD-1/PD-L1 pathway. Front. Immunol..

[9] Huang N., Fan X., Zaleta-Rivera K., Nguyen T.C., Zhou J., Luo Y., Gao J., Fang R.H., Yan Z., Chen Z.B. (2020). Natural display of nuclear-encoded RNA on the cell surface and its impact on cell interaction. Genome Biol..

[10] Flynn R.A., Pedram K., Malaker S.A., Batista P.J., Smith B.A., Johnson A.G., George B.M., Majzoub K., Villalta P.W., Carette J.E. (2021). Small RNAs are modified with N-glycans and displayed on the surface of living cells. Cell.

[11] Xie Y., Chai P., Till N.A., Hemberger H., Lebedenko C.G., Porat J., Watkins C.P., Caldwell R.M., George B.M., Perr J. (2024). The modified RNA base acp3U is an attachment site for N-glycans in glycoRNA. Cell.

[12] Li Y., Qian Y., Li X., Lei T., Monaghan-Nichols P., Fu M. (2026). Lectin-Based Detection of and Expression Profiles of Native glycoRNAs. Sci. Rep..

[13] Zhang N., Tang W., Torres L., Wang X., Ajaj Y., Zhu L., Luan Y., Zhou H., Wang Y., Zhang D. (2024). Cell surface RNAs control neutrophil recruitment. Cell.

[14] Ma Y., Guo W., Mou Q., Shao X., Lyu M., Garcia V., Kong L., Lewis W., Ward C., Yang Z. (2024). Spatial imaging of glycoRNA in single cells with ARPLA. Nat. Biotechnol..

[15] Li Y., Qian Y., Huang E., Schwarz Z., Tai H., Tillock K., Lei T., Yang X., Fu M. (2025). GlycoRNA-L and glycoRNA-S mediate human monocyte adhesion via binding to Siglec-5. Biochim. Biophys. Acta Mol. Cell Res..

[16] Graziano V.R., Porat J., Kioon M.D.A., Mejdrová I., Matz A.J., Lebedenko C.G., Chai P., Pluvinage J.V., Ricci-Azevedo R., Harrison A.G. (2025). RNA N-glycosylation enables immune evasion and homeostatic efferocytosis. Nature.

[17] Jiang X., Xu C., Yang E., Xu D., Peng Y., Han X., Si J., Shao Q., Liu Z., Chen Q. (2025). Small non-coding RNAs encapsulating mammalian cells fuel innate immunity. bioRxiv.

[18] van der Poll T., Shankar-Hari M., Wiersinga W.J. (2021). The immunology of sepsis. Immunity.

[19] Cecconi M., Evans L., Levy M., Rhodes A. (2018). Sepsis and septic shock. Lancet.

[20] Hotchkiss R.S., Moldawer L.L., Opal S.M., Reinhart K., Turnbull I.R., Vincent J.L. (2016). Sepsis and septic shock. Nat. Rev. Dis. Primers.

[21] Ge R., Jeppesen D.K., Rai S.K., Zhang Q., Higginbotham J.N., Coffey R.J., Flynn R.A. (2025). Catch and Release of sialoglycoRNAs Enables Sequencing-Based Profiling Across Cellular and Extracellular Material. bioRxiv.

[22] Jiang X., Xu C., Yang E., Xu D., Peng Y., Han X., Si J., Shao Q., Liu Z., Chen Q. (2025). Deciphering the RNA landscapes on mammalian cell surfaces. Protein Cell.

[23] Crocker P.R., Paulson J.C., Varki A. (2007). Siglecs and their roles in the immune system. Nat. Rev. Immunol..

[24] Connolly N.P., Jones M., Watt S.M. (2002). Human Siglec-5: Tissue distribution, novel isoforms and domain specificities for sialic acid-dependent ligand interactions. Br. J. Haematol..

[25] Vu H.N., Situ A.J., Dai X., Ulmer T.S. (2025). Structure of the CD33 receptor and implications for the Siglec family. Biochemistry.

[26] Montag N., Gousis P., Wittmann J. (2025). The emerging role of glycoRNAs in immune regulation and recognition. Immunol. Lett..

[27] Perr J., Langen A., Almahayni K., Nestola G., Chai P., Lebedenko C.G., Volk R.F., Detrés D., Caldwell R.M., Spiekermann M. (2025). RNA-binding proteins and glycoRNAs form domains on the cell surface for cell-penetrating peptide entry. Cell.

[28] Li Z., Joshi B.S., Yin H., Wijdeven R.H., Koç A., Zijlmans D.W., Santos-Barriopedro I., Mei H., Wu W., Shademan M. (2025). Cell-surface RNA forms ternary complex with RNA-binding proteins and heparan sulfate to recruit immune receptors. Mol. Cell.

[29] Chai P., Perr J., Kageler L., Lebedenko C.G., Dias J.M., Yankova E., Esko J.D., Tzelepis K., Flynn R.A. (2024). Cell surface ribonucleoproteins cluster with heparan sulfate to regulate growth factor signaling. bioRxiv.

[30] Zhang Z., Ling T., Ding Q., Zhu F., Cheng X., Li X., Ma T., Meng Q. (2025). GlycoRNA-rich, neutrophil membrane-coated, siMT1-loaded nanoparticles mitigate abdominal aortic aneurysm progression by inhibiting neutrophil extracellular trap formation. Mater. Today Bio.

[31] Abledu J.K., Herbst C.J., Brandt R., Kocak A., Ghosh P., López J.L.G., Diestelhorst K., Block S., Hackenberger C., Seitz O. (2025). Cell surface RNA expression modulates alveolar epithelial function. Am. J. Respir. Cell Mol. Biol..

[32] Feinberg E.H., Hunter C.P. (2003). Transport of dsRNA into cells by the transmembrane protein SID-1. Science.

[33] Ren T., Zhang Y., Tong Y., Zhang Q., Wang T., Wang Y., Yang C., Xu Z. (2025). FRET imaging of glycoRNA on small extracellular vesicles enabling sensitive cancer diagnostics. Nat. Commun..

[34] Gong Z., Yuan P., Gan Y., Tang Y., Li K., Liu Z., Wang Y., Zhong S., Yang Y., Qing Z. (2025). Intramolecular proximity-induced amplification for accurate imaging of glycosylated RNAs in living cells and zebrafish. Anal. Chem..

[35] Brunner C.M., Pietsch L., Sondern I.V., Röhrl M., Popov C., Trollmann M.F., Taylor R.W., Blessing M., Holler C., Almahayni K. (2025). Bottom-up investigation of spatiotemporal glycocalyx dynamics with interferometric scattering microscopy. J. Am. Chem. Soc..

[36] Ge J., Han J., Fang X., Wang C., Yang Y. (2025). Comprehensive and facile strategy for enhanced visualization of sialylated RNA via dual bioorthogonal labeling. ACS Chem. Biol..

[37] Hazemi M.E., Geeson M.B., Müller F.M., Mikutis S., Enright A.J., Bernardes G.J.L. (2025). An expanded view of RNA modification with carbohydrate-based metabolic probes. JACS Au.

